# Retinal sensitivity and fundus autofluorescence in adult-onset foveomacular vitelliform dystrophy

**DOI:** 10.1038/s41598-023-49256-1

**Published:** 2023-12-08

**Authors:** Ryosuke Fujino, Tatsuya Inoue, Yasuo Yanagi, Maiko Maruyama-Inoue, Kazuaki Kadonosono, Ryo Obata, Ryo Asaoka

**Affiliations:** 1 Department of Ophthalmology, The University of Tokyo, Tokyo, Japan; 2 Department of Ophthalmology and Micro-Technology, Yokohama City University, 4-57 Urafune, Minami-ku, Yokohama, 232-0024 Japan; 3 Department of Ophthalmology, Seirei Hamamatsu General Hospital, 2-12-12 Sumiyoshi, Naka-ku, Hamamatsu, Shizuoka 430-8558 Japan; 4 Seirei Christopher University, Hamamatsu, Shizuoka Japan

**Keywords:** Macular degeneration, Vision disorders

## Abstract

The present study aimed to compare retinal sensitivity (RS) at each stage and to evaluate the relationship between RS and fundus autofluorescence (FAF) pattern in adult-onset foveomacular vitelliform dystrophy (AOFVD). We retrospectively reviewed 17 eyes of 13 patients with AOFVD. In addition to best-corrected visual acuity (VA), RS within the affected lesion and optical coherence tomography (OCT) measurements were carried out in each participant. All the examined eyes were classified into 4 stages and 3 FAF patterns. RS was superimposed on OCT fundus image and RS within the affected lesion was calculated in each eye. The relationships between visual functions (VA and RS within the affected lesion) and stages and also FAF patterns were analyzed using the linear mixed model. As a result, RS within the affected lesion was significantly associated with FAF pattern, but not with stage. In contrast, VA was correlated with neither stages nor FAF patterns. Our current result suggested that RS within the affected lesion was related to FAF patterns but this was not the case with VA in eyes with AOFVD, demonstrating the usefulness of measuring RS, not only VA, to comprehend the disease status in AOFVD.

## Introduction

Adult-onset foveomacular vitelliform dystrophy (AOFVD) is a subtype of macular pattern dystrophies which usually occurs at the age of between 30 and 50 years, in contrast to the typical Best vitelliform macular dystrophy (VMD) which generally emerges during childhood. AOFVD can progress even in a relatively short period (10% in 16.2 months in average)^1^; yellowish subretinal vitelliform material is accumulated in the macula, which progresses over time and eventually transforms to an atrophic lesion. Therefore, AOFVD is classified into the following 4 stages, as originally proposed for VMD: (1) vitelliform, (2) pseudohypopyon, (3) vitelliruptive and (4) atrophic stages^1^. There are previous studies suggested that retinal sensitivity (RS) was deteriorated according to the progression of the disease stage in VMD^2^ or AOFVD^3^. On the other hand, Parodi et al. proposed a classification of the fundus autofluorescence (FAF) patterns; hyper FAF, hypo FAF and patchy FAF patterns in VMD, and suggested visual acuity (VA) and RS were deteriorated especially in eyes with hypo FAF pattern^4^. However, it is also true that different finding was also reported; another previous study reported retinal sensitivities in eyes with patchy FAF pattern were worse compared to the eyes with hyper FAF pattern in AOFVD^5^. Thus, it has remained unclear how visual function differs across FAF patterns in AOFVD. It is also important that this association should be investigated in conjunction with disease stage, because they are correlated to each other, however the relationship between disease stage and FAF pattern was not investigated in these previous studies.

To shed light on this issue, we measured RS within the affected lesion and investigated the relationship between RS within the affected lesion and disease stages and also FAF patterns in AOFVD eyes. The MP-3 microperimeter (Nidek co.ltd, Aichi, Japan) was used to evaluate RS in detail, because the deteriorated central visual function may raise a fixation problem during the visual field (VF) measurement^6–8^.

## Results

The mean (± standard deviation: SD) age was 67.9 ± 12.1 [range 51–86] years, and 9 patients were males and 4 patients were females. Best corrected visual acuity (BCVA) was 0.12 ± 0.21 [− 0.079 to 0.70] logarithm of the minimum angle of resolution (logMAR), RS within the affected lesion was 19.1 ± 6.8 [2.3–27] dB.

As shown in Table 1, there were 5 eyes (29.4%) in the vitelliform stage, 3 eyes (17.7%) in the pseudohypopyon stage, 5 eyes (29.4%) in the vitelliruptive stage, 4 eyes (23.5%) in the atrophic stage. 3 eyes (17.7%), 10 eyes (58.8%), and 4 eyes (23.5%) showed hyper FAF, patchy FAF, and hypo FAF patterns respectively, as shown in Table 2.

**Table 1:** Clinical characteristics in AOFVD patients with each disease stage.

Disease stage	N of eyes (%)	N of hyper FAF (%)	N of patchy FAF (%)	N of hypo FAF(%)	BCVA	RS (dB)	RV (mm^3^)	CCT (μm)
Vitelliform	5 (29.4)	2 (40)	3 (60)	0 (0)	0.053 ± 0.12	23.9 ± 2.3	2.4 ± 0.083	253.4 ± 57.3
Pseudohypopyon	3 (17.1)	0 (0)	3 (100)	0 (0)	0.0993 ± 0.13	22.5 ± 2.9	2.4 ± 0.14	197.7 ± 16.7
Vitelliruptive	5 (29.4)	1 (20)	4 (80)	0 (0)	0.046 ± 0.093	21.3 ± 2.	2.3 ± 0,36	282.4 ± 31.2
Atrophic	4 (23.5)	0 (0)	0 (0)	100 (100)	0.33 ± 0.29	8.0 ± 3.4	2.2 ± 0.15	221.3 ± 74.9

*FAF* fundus autofluorescence, *BCVA* best-corrected visual acuity, *RS* retinal sensitivity, *RV* retinal volume, CCT: central choroidal thickness.

**Table 2:** Clinical characteristic in AOFVD patients with each FAF pattern.

FAF pattern	No of eyes (%)	BCVA	RS (dB)	RV (mm^3^)	CCT (μm)
Hyper	3 (17.7)	0.021 ± 0.14	22.9 ± 2.0	2.3 ± 0.090	304.3 ± 45.7
Patchy	10 (58.8)	0.073 ± 0.10	22,4 ± 2.9	2.3 ± 0.27	235.9 ± 43.1
Hypo	4 (23.5)	0.33 ± 0.29	7.96 ± 3.4	2.2 ± 0.15	221.3 ± 74.9

*FAF* fundus autofluorescence, *BCVA* best-corrected visual acuity, *RS* retinal sensitivity.

*RV* retinal volume, *CCT* central choroidal thickness.

**Figure 1:**
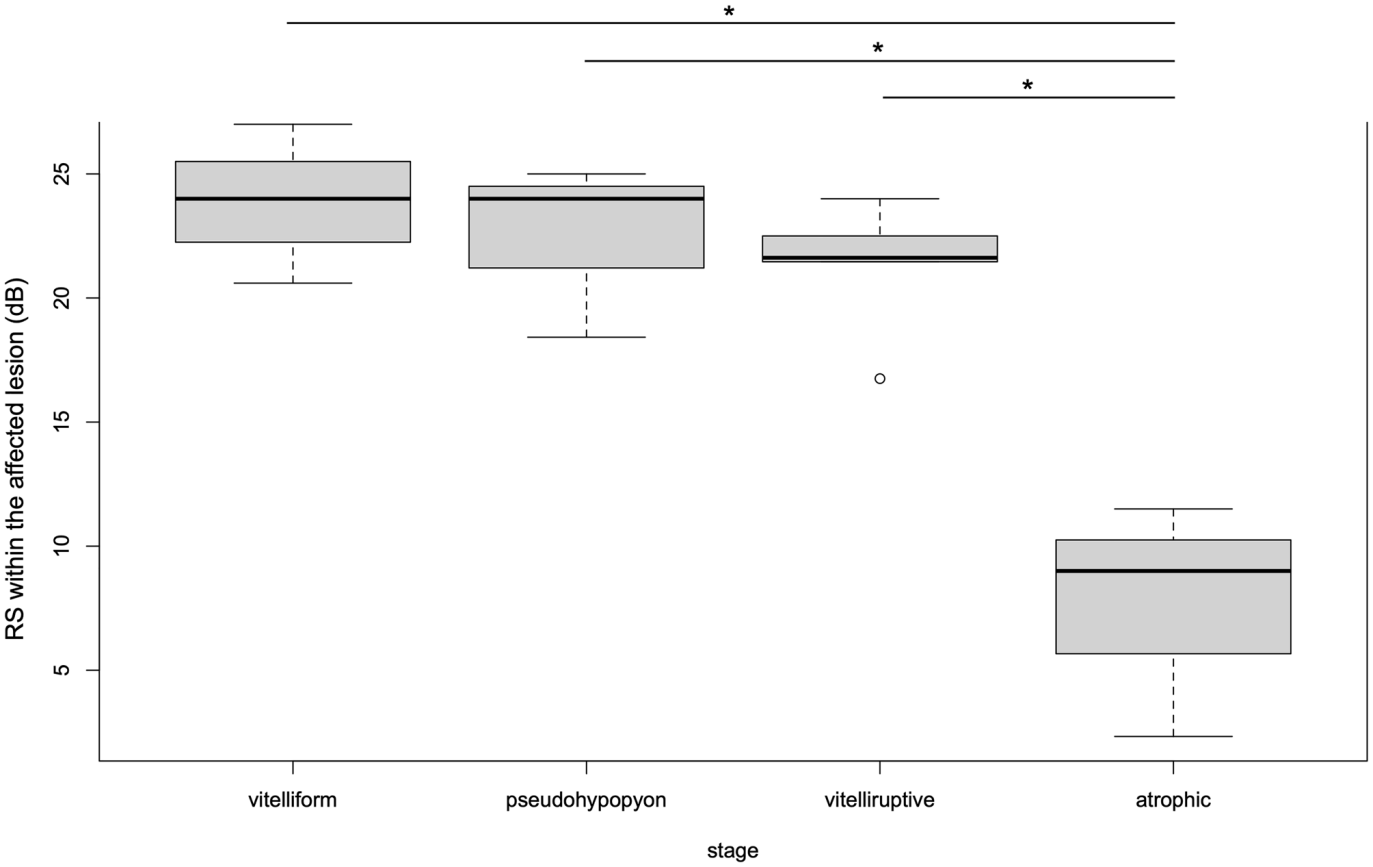
Boxplot of RS within the affected lesion of each stage. RS within the affected lesion was significantly decreased in eyes at atrophic stage compared with other stages (*p < 0.001, ANOVA, respectively) using linear mixed regression model. RS: retinal sensitivity.

RS within the affected lesion in the atrophic stage was significantly decreased than those in vitelliform, pseudohypopyon, and vitelliruptive stages (*p* < 0.001, respectively, linear mixed regression model). On the other hand, there was no significant difference across other 3 stages of vitelliform, pseudohypopyon, and vitelliruptive (Fig. 1).

**Figure 2:**
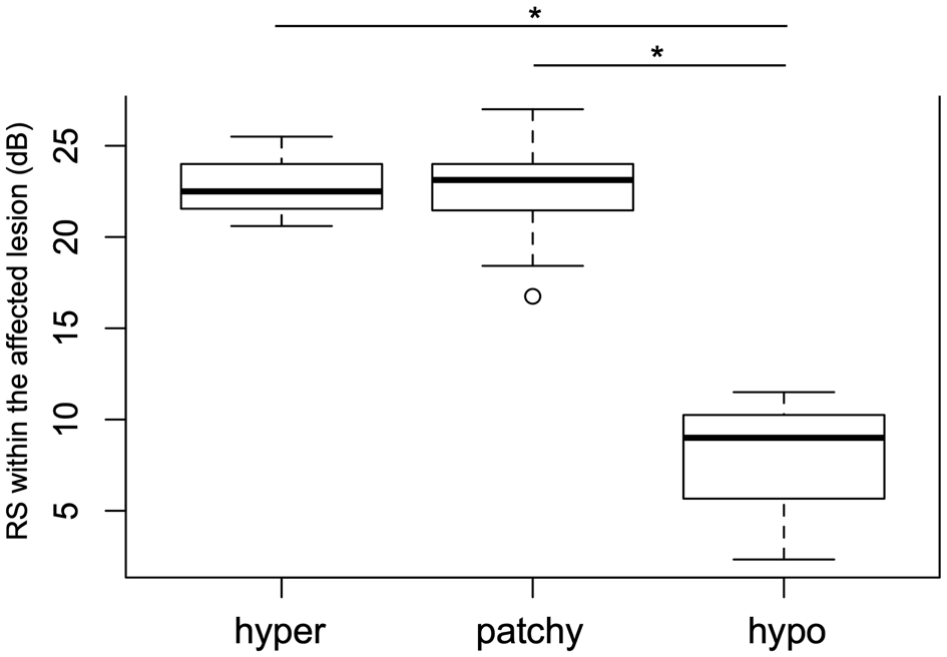
Boxplot of RS within the affected lesion of each FAF pattern. RS within the affected lesion was significantly decreased in eyes with hypo-autofluorescent pattern compared with hyper-autofluorescent and patchy pattern (*p < 0.001, ANOVA, respectively) using linear mixed regression model. RS: retinal sensitivity, hyper: hyper-autofluorescent pattern, patchy: patchy pattern, hypo: hypo-autofluorescent pattern.

RS within the affected lesion was significantly declined in eyes with hypo-FAF pattern, compared to eyes with hyper and patchy FAF patterns (*p* < 0.001, respectively), although there was no significant difference between hyper and patchy FAF patterns (*p* = 0.85, linear mixed regression model, Fig. 2).

As a result of AICc model selection for RS within the affected lesion, only FAF pattern was selected in the optimal model, and the remaining variables of disease stage, IOL status and age were not selected, as shown below;

RS within the affected lesion (dB) = 8.0 + 14.8 × hyper FAF pattern (SE = 2.4, *p* < 0.001) + 14.4 × patchy FAF pattern (SE = 1.9, *p* < 0.001) (AICc = 100.0).

The optimal model for BCVA by the AICc model selection method had no variable selected.

The relationship between disease stage and FAF pattern was investigated using the linear mixed regression model. As a result, there were significant differences in disease stage between eyes with hyper-FAF pattern and hypo-FAF pattern, and between patchy FAF pattern and hypo-FAF pattern (*p* < 0.001, linear mixed model, respectively).

## Discussion

In the current study, we investigated the relationships between visual functions (RS and BCVA) and disease stage and FAF pattern in eyes with AOFVD. As a result, RS within the affected lesion was significantly correlated with FAF pattern, but not with disease stage, when FAF pattern and disease stage were investigated simultaneously. On the other hand, BCVA was not correlated with either disease stage or FAF pattern. This would imply that RS within the affected lesion reflects retinal function more sensitively than BCVA in AOFVD eyes.

There have been studies investigated RS at various stages in eyes with Best disease and also AOFVD. Battaglia et al. have reported that RS was significantly reduced at atrophic stage compared to all other stages, whereas there was no significant difference across other 3 stages of vitelliform, pseudohypopyon, and vitelliruptive stages in eyes with Best disease^2^. Grenga et al.^3^ indicated that a significant reduction was observed in RS at atrophic stage compared to pseudohypopyon and vitelliruptive stages in AOFVD eyes. We also compared RS across disease stage, and it was consistent with these previous results in general. Nonetheless, current results suggested that there was a close correlation between disease stage and FAF pattern and as a result, FAF pattern was included in the optimal model for RS, but disease stage was not. This implies FAF pattern is more useful than disease stage when analyzing RS in AOFVD eyes.

There have been studies suggested the impact of FAF pattern on RS in AOFVD; Parodi et al.^5^ reported in SW-AF image of AOFVD patients, eyes with patchy FAF pattern showed worse RS than those with hyper-FAF pattern. Another study^4^ also suggested that RS was significantly decreased in eyes with patchy and hypo-FAF patterns compared to hyper-FAF pattern in SW-AF image of Best disease patients. Our results coincided with these results, however different in that there was a significant difference in RS between eyes with hyper and patchy FAF patterns in these previous studies. The entire reason for these contracting results is not clear, however this may be attributed to the differences of the device to measure RS. These previous studies used the MP-1 microperimeter (Nidek Co. Ltd, Aichi, Japan) to calculate RS, but we used the MP-3. In contrast to the MP-1, the MP-3 has a wide dynamic range (between 0 and 34 dB) on the background luminance of 31.5 ASB which is identical to that with the HFA. In addition, in the current study, RS at the affected retinal lesion was precisely measured, whereas the RS in a fixed area (2, 4, 6, or 10 degrees around fovea) was used in the previous studies^4,5^ (i.e., without identifying the affected retinal lesion), and hence RS in unaffected lesion was also included.

Regarding the association between BCVA and disease stage, Battaglia et al.^2^ reported that BCVA was significantly poor at atrophic stage compared to all other stages, whereas there was no significant difference in the value across vitelliform, pseudohypopyon, and vitelliruptive stages in eyes with Best disease. Another previous study reported BCVA was better at vitelliform stage compared to pseudohypopyon and vitelliruptive stages in eyes with patchy FAF pattern in Best disease^4^. Regarding the association between BCVA and FAF pattern, a previous study reported that BCVA was significantly poor in eyes with patchy FAF pattern compared to hyper-FAF pattern in patients with AOFVD (hypo-FAF pattern was not included in the analysis)^5^. Another previous study suggested that there was a significant difference in BCVA between hyper-FAF pattern and patchy or hypo-FAF patterns in eyes with Best disease, but not between patchy and hypo-FAF patterns^4^. On the other hand, in the current study, BCVA was not significantly correlated with either disease stage or FAF pattern as a result of multivariate analysis followed by the AICc model selection. The entire reason for the different results between the current study and these previous studies is unclear. It may be recommended to conduct a further confirmatory study preparing samples in a larger size. In contrast, in the current study, FAF pattern was included in the optimal model for RS within the affected lesion, implying that RS within the affected lesion reflected retinal function more sensitively than BCVA in AOFVD eyes.

Our study included several limitations. First, this was a retrospective cross-sectional analysis with a relatively small sample size, and the genetic examination was not performed; we diagnosed AOFVD according to the age of onset and clinical examination findings consistent with AOFVD. Secondly, the integrity of the ellipsoid zone (EZ) was not taken into consideration in our present study. Freund and associates recently reported that BCVA was correlated with the degree of EZ integrity in patients with acquired vitelliform lesions^9^. We have reported a novel method for quantifying residual EZ index in macular diseases, such as CSC or cone-rod dystrophy^10,11^. It would be of interest to further investigate the relationships between the EZ integrity and visual function in AOFVD eyes.

In conclusion, RS within the affected lesion was significantly related to FAF pattern in eyes with AOFVD, whereas BCVA was not correlated with either disease stage or FAF pattern. Therefore, RS within the affected lesion was more sensitive than BCVA in evaluating retinal function in AOFVD eyes. Disease stage had a weaker correlation with RS than FAF pattern.

### Ethical approval

All procedures performed in studies involving human participants were in accordance with the ethical standards of the institutional and/or national research committee and with the 1964 Helsinki declaration and its later amendments or comparable ethical standards.

### Informed consent

Informed consent was obtained from all individual participants included in the study.

## Method

This retrospective and cross-sectional study was approved by the Research Ethics Committee of the Graduate School of Medicine and Faculty of Medicine at The University of Tokyo (#2217). Written consent was given by all patients for their information to be stored in the hospital database and used for research. This study was performed according to the tenets of the Declaration of Helsinki.

### Subjects

This study included 17 eyes of 13 patients (9 male and 4 females) diagnosed as AOFVD as follows: the age of onset was older than 30 years old; complaint of visual impairment or metamorphopsias; round or heterogenous yellowish or atrophic macular lesions in the fundus examinations. Exclusion criteria were: a history of other retinal disease such as age-related macular degeneration, epiretinal membrane or macular hole or any other ocular disease that could impair vision such as amblyopia, glaucoma, and optic neuropathy, or a history of intraocular surgery within 6 months. All patients underwent comprehensive ophthalmic examinations, including BCVA, VF, FAF, and spectral domain OCT. AOFVD patients were divided into 4 stages using multimodal fundus imaging based on the previous report^1^; (I) vitelliform stage; (II) pseudohypopyon stage; (III) vitelliruptive stage; and (IV) atrophic stage.

### Optical coherence tomography measurement

OCT measurement was carried out using the Spectralis OCT (Heidelberg Engineering, Heidelberg, Germany). The OCT images consisted of line scans (horizontal and vertical B-scans) and raster scans. Line scans were created by taking the average of 100 B-scans (768 A-scans per B-scan) within 30°. The raster scan was performed using 25 B-scans (768 A-scans per B-scan) of a 30° × 20° area.

**Figure 3:**
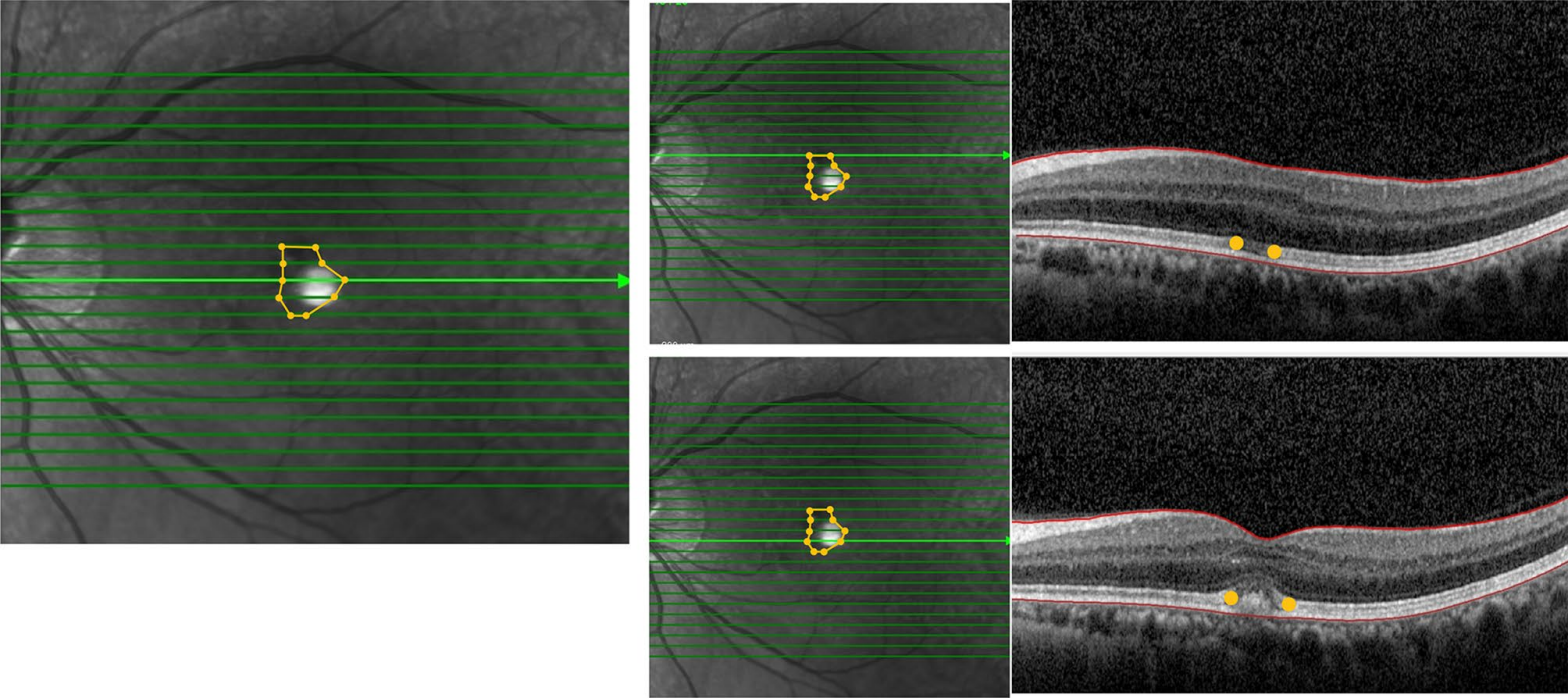
OCT images of the affected lesion in a represent case (52 years old, male). The boundaries between normal and affected area in fovea were plotted on a horizontal OCT scan. The affected lesion is expressed connecting yellow dots of all of the slices.

The affected area was identified using either of reflective materials (hyperreflective, hyporeflective, or mixed hyperreflective and hyporeflective) or disrupted / absence of ellipsoid zone (EZ) in fovea on the OCT image. The boundaries between normal and affected area were detected and plotted on a horizontal OCT scan (Fig. 3).

The affected lesion was initially identified by one examiner (R.F.), followed by another independent examiner (T.I.) without reviewing the results of the first examiner. If the second estimator did not agree with the first examiner, a panel discussion (R.F., T.I., R.O.) was held to draw a conclusion.

Retinal volume (RV) within a 3 mm diameter and central choroidal thickness (CCT) were calculated using the Spectralis OCT. RV was calculated based on the thickness between the inner limiting membrane and Bruch’s membrane.

### MP-3 measurement

RS were measured using the MP-3 microperimetry. With MP-3, the position of the retina is automatically tracked and the location of the target is aligned accordingly. As a result, the retinal location stimulated at each target presentation is precisely determined, which would be advantageous to evaluate RS in detail in AOFVD, because the deteriorated central visual function may raise a fixation problem during the VF measurement^6–8^. All patients had a pupil size larger than 4 mm in diameter, as required for the MP-3 measurement. The MP-3 measurement was carried out using the 4–2 full threshold staircase strategy using the standard Goldmann III stimulus size on the background luminance of 31.4 asb. The maximum luminance of the MP-3 is 10,000 asb, which results in the stimulus dynamic range between 0 and 34 dB. Eccentric circle was drawn at 2, 4 and 6 degrees from fovea and eight test points were allocated on each circle, in addition to fovea. Only reliable VFs, defined as fixation loss (FL) rate < 20% and a false-positive (FP) rate < 15%, were used in analyses, similarly to the recommendation for the Humphrey Field Analyzer (HFA; CARL ZEISS Meditec, Dublin, California, USA).

### RS within the affected lesion

**Figure 4:**
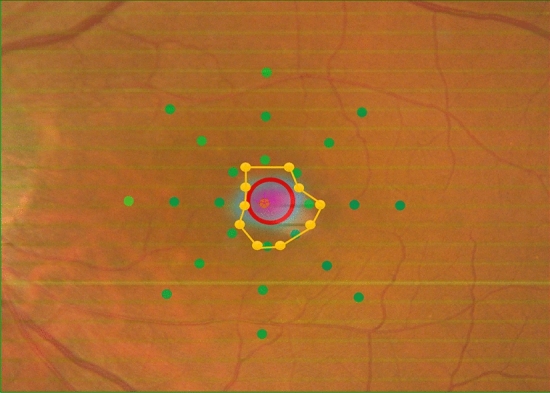
MP-3 image superimposed onto an OCT image in a represent case (52 years old, male). The mean retina sensitivity within the affected lesion expressed with Fig. 1 indicating method was calculated as RS within the affected lesion.

RS were measured using the MP-3 microperimetry at 25 points within the central 12-degrees around fovea. Subsequently, the affected lesion was identified using the Spectralis OCT. The OCT image was superimposed on MP-3 image using the ImageJ software (http://imagej.nih.gov/ij/; provided in the public domain by the National Institutes of Health, Bethesda, MD, USA) and then RS within the affected lesion was calculated as the mean RS within the affected lesion (Fig. 4).

### FAF image

**Figure 5:**
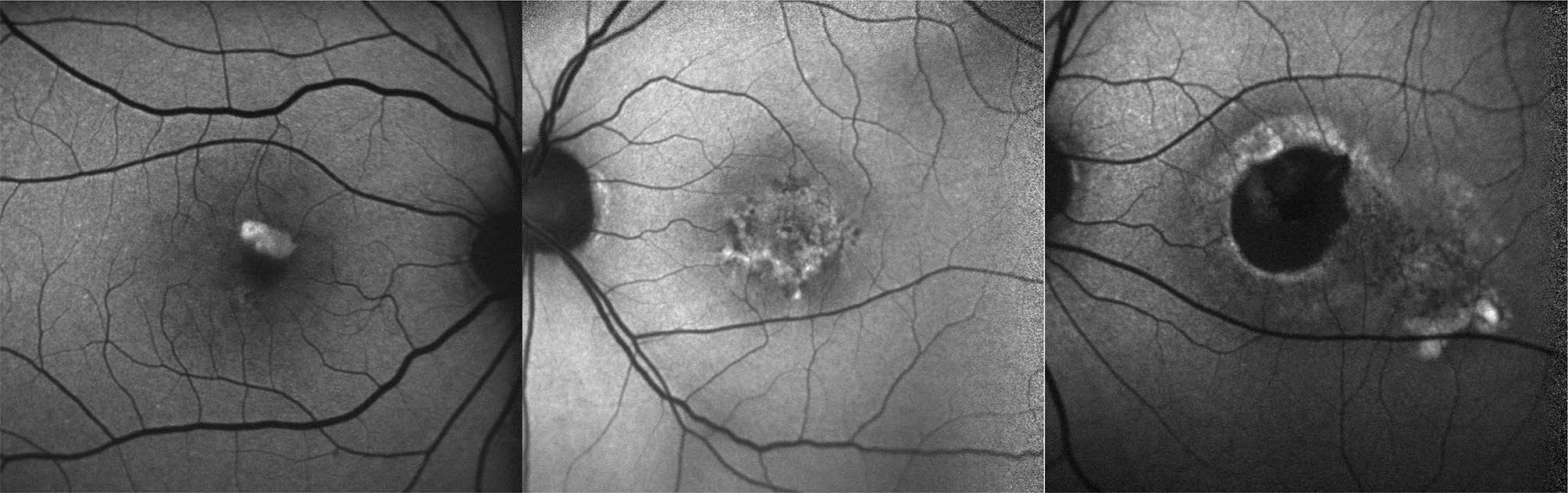
Three FAF patterns. Three FAF patterns were identified on FAF image within central 30 × 30 degrees area. (Left) hyper-autofluorescent pattern, (middle) patchy pattern and (Right) hypo-autofluorescent pattern.

FAF image was obtained using a short-wavelength AF (SW-AF) (HRA2; Heidelberg Engineering, Heidelberg, Germany) within central 30 × 30 degrees from fovea with a resolution of 768 × 768 pixels. For the SW-AF imaging, a wavelength of 488 nm was used for excitation, and the emitted light was above 500 nm and detected through a barrier filter. Based on a previous study^4^, FAF patterns were classified into 3 groups: hyper-FAF pattern (increased foveal FAF signal), patchy FAF pattern (combination of increased and decreased foveal FAF signal) and hypo-FAF pattern (decreased foveal FAF signal) (Fig. 5).

### Statistical analysis

(1) The relationship between RS within the affected lesion and disease stage, FAF pattern, IOL status and age was investigated using the linear mixed model, whereby the data was nested within patients. The linear mixed model is equivalent to ordinary linear regression in that the model describes the relationship between the predictor variables and a single outcome variable; however, standard linear regression analysis makes the assumption that all observations are independent of each other. In the current study, measurements were nested within subjects and, thus, dependent on each other. Ignoring this grouping of the measurements would result in an underestimation of standard errors of the regression coefficients. The linear mixed model adjusts for the hierarchical structure of the data, modeling in a way in which measurements are grouped within subjects to reduce the possible bias of including both eyes of one patient^12,13^. Followingly, the optimal model for RS within the affected lesion was identified according to the second order bias corrected Akaike Information Criterion (AICc) index. The AICc is the corrected form of the common statistical measure of AIC. AICc gives an accurate estimation even when the sample size is small^14^. In a multivariate regression model, the degree of freedom decreases as the number of variables increases, hence it is recommended to use model selection methods to improve the model by removing redundant variables^15,16^. (2) Similar analyses were conducted for the relationship between BCVA and disease stage, FAF pattern, IOL status and age were investigated using the linear mixed model. (3) In addition, the relationship between disease stage and FAF pattern was investigated using the linear mixed model. All statistical analyses were performed using the statistical programming language ‘R’ (R version 3.4.3; e foundation for Statistical Computing, Vienna, Austria).

## Data Availability

The datasets used and analyzed during the current study available from the corresponding author on reasonable request.
